# Determinants of an elevated pulmonary arterial pressure in patients with pulmonary arterial hypertension

**DOI:** 10.1186/s12931-015-0246-y

**Published:** 2015-07-08

**Authors:** Seiichiro Sakao, Norbert F. Voelkel, Nobuhiro Tanabe, Koichiro Tatsumi

**Affiliations:** Department of Respirology (B2), Graduate School of Medicine, Chiba University, 1-8-1 Inohana, Chuo-ku, Chiba 260-8670 Japan; Victoria Johnson Center for Obstructive Lung Diseases and Pulmonary and Critical Care Medicine Division, Virginia Commonwealth University, Molecular Medicine and Research Building, Richmond, VA 23298-0456 USA

**Keywords:** Pulmonary arterial hypertension (PAH), Pulmonary vascular remodeling, Heath-edwards classification

## Abstract

Given the difficulty of diagnosing early-stage pulmonary arterial hypertension (PAH) due to the lack of signs and symptoms, and the risk of an open lung biopsy, the precise pathological features of presymptomatic stage lung tissue remain unknown. It has been suggested that the maximum elevation of the mean pulmonary arterial pressure (*P*_pa_) is achieved during the early symptomatic stage, indicating that the elevation of the mean *P*_pa_ is primarily driven by the pulmonary vascular tone and/or some degree of pulmonary vascular remodeling completed during this stage. Recently, the examination of a rat model of severe PAH suggested that the severe PAH may be primarily determined by the presence of intimal lesions and/or the vascular tone in the early stage. Human data seem to indicate that intimal lesions are essential for the severely increased pulmonary arterial blood pressure in the late stage of the disease.

However, many questions remain. For instance, how does the pulmonary hemodynamics change during the course of the disease, and what drives the development of severe PAH? Although it is generally acknowledged that both pulmonary vascular remodeling and the vascular tone are important determinants of an elevated pulmonary arterial pressure, which is the root cause of the time-dependent progression of the disease? Here we review the recent histopathological concepts of PAH with respect to the progression of the lung vascular disease.

## Introduction

Almost all of the previous large studies which have described the features of pulmonary vascular remodeling in patients with severe angioobliterative PAH, including IPAH and PAH associated with congenital heart disease (CHD), have been based on autopsy samples obtained from patients with end stage PAH [1]. Given the difficulty of diagnosing early-stage PAH because of the lack of signs and symptoms [2–5], and because we lack open lung biopsies that are risky in patients with severe PAH, the pathological features of early lung vascular disease (during the presymptomatic stage) in patients with severe PAH remain unclear. Most likely severe angioobliterative PAH is not histologically homogeneous and the pathological grading method proposed by Heath and Edwards to assess PAH is based largely on lung tissue samples from patients with CHD [6]. Heath and Edwards demonstrated that, among PAH patients with CHD, the medial thickness of the muscular pulmonary arteries and muscularization of the pulmonary arterioles without intimal alterations (grade 1) are the earliest abnormalities of the pulmonary vasculature. These patients were subjected to high systemic blood pressure from birth due to congenital cardiac anomalies. Moreover, they showed that, together with the medial hypertrophy, intimal thickness with cell proliferation in the smaller muscular pulmonary arteries (grade 2), intimal obstruction with concentric or eccentric masses of less cellular fibrous tissue (grade 3) and progressive (grade 4) and chronic (grade 5) dilation of the small arteries with plexiform and angiomatoid lesions, appear as a result of an elevated blood pressure in the pulmonary arteries due to CHD [6] (Table 1). Although never supported by evidences, it is generally believed that these pathological alterations gradually progress over time from grade 1 to 5 [6].

**Table 1:** The Heath and Edwards pathological grading method

Potenially Reversable	
1	The medial thickness of the muscular pulmonary arteries and muscularization of the pulmonary arterioles without intimal alterations.
2	Together with the medial hypertrophy, intimal thickness with cell proliferation in the smaller muscular pulmonary arteries.
3	Intimal obstruction with concentric or eccentric masses of less cellular fibrous tissue in the arterioles and small muscular arteries. Large elastic arteries show atherosclerosis.
Usually Irreversible	
4	Progressive dilatation of the small arteries with plexiform lesions and muscle hypertrophy is less apparent.
5	Chronic dilatation of the small arteries with plexiform and angiomatoid lesions.
6	Necrotizing arteritis with thrombosis.

(Heath D et al., Circulation 1958, 18: 533-547)

It has also been suggested that the maximum elevation of the mean pulmonary arterial pressure (*P*_pa_) in PAH patients is achieved during the early symptomatic stage [2, 4], indicating that the elevation of the mean *P*_pa_ is driven by the pulmonary vascular tone and/or the degree of pulmonary vascular remodeling completed during this stage. The relative contribution of vasoconstriction to the mean *P*_pa_ varies between patients [7] and is perhaps larger during the presymptomatic phase of PAH due to the progression of pulmonary vascular remodeling in a time-dependent manner [6].

Recently, in a rat model of severe PAH it has been shown that the elevated pulmonary arterial pressure appears to be primarily driven by grade 1 and 2 remodeling (according to the Heath-Edwards classification) and/or the vascular tone. Complex vascular lesions, including those of Heath-Edwards grade 4, may develop as a consequence of the high pulmonary arterial pressure or shear stress [8, 9].

Here we review the recent histopathological concepts of PAH with respect to the stage of disease progression, guided by the questions: how does pulmonary hemodynamics change during the course of the disease and what degree of pulmonary vascular remodeling increases the *P*_pa_? We first mention the recent pathophysiological findings in a rodent model of severe PAH and then the histopathological features in patients with PAH who had been treated with the currently available PAH-targeted drugs. Subsequently, we consider the determinants of the elevated pulmonary arterial pressure from these findings and the therapeutic strategies in accordance with different disease stages.

## Alterations of the pulmonary hemodynamics during the course of the disease according with Heath-Edwards classification: lessons from the “Sugen/Hypoxia” animal model of pulmonary hypertension

It has been reported that in a rat model of severe PAH plexiform-like lesions [8, 10], characteristic of advanced PAH, develop [11]. This model (the Sugen/Hypoxia model) is based on a single percutaneous injection in rats of a vascular endothelial growth factor receptor blocker (SUGEN5416) which is combined with chronic exposure to hypoxia for three weeks. As classical rodent models of mild to moderate pulmonary hypertension (PH), the chronic hypoxia and monocrotaline models have been used to investigate the mechanistic basis for the development of PH [12]. However, they lack phenotypically altered proliferated endothelial cells (ECs) in the lumen of pulmonary arteries and their defining pulmonary vascular remodeling was medial muscular thickening of proliferating smooth muscle cells (SMC) [13]. Therefore, there has been no obvious report focusing on the Heath-Edwards classification in these rodent models. On the other hand, in this model, not only plexiform-like lesions, but also all the pulmonary vascular abnormalities described in the Heath-Edwards classification, develop in a time-dependent manner. Importantly there is also a linear correlation between the right ventricular (RV) systolic pressure (RVSP) and the number of obliterated vessels [14]. Remarkably, although the rats in this model develop severe PAH at five weeks after the SUGEN5416 injection, only grade 1 and 2 lesions of the Heath-Edwards classification were identified at this time point. This suggests that the severely elevated pulmonary arterial pressure observed in this rat model is due to these grade 1 and 2 lesions and/or the vascular tone increase in the early stage of the disease. The intimal occlusive lesions gradually progress in this rat model while the degree of medial thickness is decreasing [15]. These results suggest that a Heath-Edwards grade greater than 2, i.e., intimal lesions rather than medial lesions, appear to be mainly responsible for the increased pulmonary arterial blood pressure and the increased pulmonary arterial resistance (PVR) during the later stages of the disease. More recently de Raaf et al. used telemetry to monitor the time course of the increase in the RVSP [9] and found a reversible hypoxic vasoconstriction component in the Sugen/Hypoxia model and progressive intima remodeling and a media muscularization that was in proportion to the chronic hypoxia challenge.

The authors postulated that the hemodynamic and histological progression in this model are linked, however it has been critically acknowledged that animal models may not fully develop the complete spectrum of human histopathology, particularly when it comes to the time-dependence of the vascular changes [16]. The Sugen/Hypoxia rats are kept in a hypoxic chamber for the first three weeks of the protocol, indicating that chronic hypoxic pulmonary vasoconstriction (HPV) [17] likely induces high shear stress [18] and increased generation and secretion of growth factors and inflammatory cytokines within and around the pulmonary vasculature. This is associated with the appearance of smooth muscle-like cells [19]. In human diseases, however, prolonged alveolar hypoxia likely occurs only in highlanders with PH [20] and regionally in patients with respiratory disease, including chronic obstructive pulmonary disease (COPD) and pulmonary fibrosis [21]. Taking this fact into account, the chronic hypoxia PAH model cannot reproduce the complete human pathobiology. In human forms of severe PAH phenotypically altered endothelial cells (ECs) [22, 23], uncontrolled cell proliferation [18, 24], inflammation [25, 26] and factors which can cause vasoconstriction or vasodilation [27–29], all appear to have a role in increasing the pulmonary vascular resistance.

## Sequential vascular remodeling according to the Heath-Edwards classification in human PAH

Stacher and coworkers recently performed a detailed analysis of the histopathology of explanted lung samples obtained from 62 PAH patients and 28 control subjects in order to investigate the potential effects of modern therapy (prostacyclin and its analogs, phosphodiesterase type 5 inhibitors and endothelin receptor blockers) on the histopathological features observed in these patients. The authors demonstrated that a considerable number of the explanted lung samples from patients with endstage PAH, who had been treated with modern PAH therapy, exhibited medial thickening that overlapped with the degree of media thickening found in the control lungs. On the other hand, they had a similar number of plexiform lesions as patients with other forms of progressive PAH [1]. Of the PAH patients 48 were characterized as a group with an IPAH-like pattern, which included 27 patients with IPAH, 9 patients with CHD-PAH, 5 patients with familial PAH, 3 patients with drug-related PAH, 2 patients with venoocclusive disease, 1 patient with collagen vascular disease-PAH, and 1 patient with chronic thromboembolic pulmonary hypertension. There were no significant differences in the morphometric measurements or the plexiform lesions between the subgroups.

One particularly important result derived from the morphometric analysis of the PAH lesions in this study [1] is that sequential alterations in pulmonary vascular remodeling, as proposed by Heath and Edwards, may not be present (or may not occur) in PAH patients treated with modern therapy. The authors found plexiform lesions in the IPAH and associated PAH patients in spite of unimpressive medial remodeling. A subset of the explanted lung samples obtained from PAH patients with a progressive stage of disease had rather unremarkable medial lesions [1]. This fact supports the finding that Heath-Edwards classification grades 2 and/or 3 are likely responsible for the increased pulmonary arterial blood pressure in the Sugen/Hypoxia model [15]. At the time of lung transplantation, the intimal lesions with phenotypically altered mesenchymal-like cells [18, 22, 28, 29], including plexiform lesions, rather than medial remodeling, appeared to determine the elevated *P*pa. However, there was no correlation between the number of plexiform lesions and the pulmonary hemodynamic values [1, 30] and there was only a tendency for a correlation between the intima plus media fractional thickness and hemodynamic values [1]. Thus, taken together, this suggests that, if the degree of occluded and stenosed pulmonary arterioles may determine the hemodynamics in patients with advanced PAH, similar to the Sugen/Hypoxia model where there is a strong correlation between the RVSP and vessel lumen obliteration [14], the degree of these vascular remodeling may not be accurately assessed by either the intimal fractional thickness, the intimal volume density or the number of plexiform lesions.

Alternatively, or additionally still to be identified vasoconstrictive substances may contribute to the increased *P*pa, even in end stage patients prior to lung transplantation [14]. However, at this stage a thinned and fibrosed media of the muscular arteries and arterioles appears [6], making it less likely that severe vasoconstriction dominates the picture.

If vasoconstriction is prominent, as it must be in those patients who are treatable with calcium channel blockers, it is of interest that these patients do not progress. We postulate that there is likely no pulmonary angioobliteration. However, histopathological studies of these patients’ lungs are lacking.

## Determinants of the elevated pulmonary arterial pressure

**Fig. 1:**
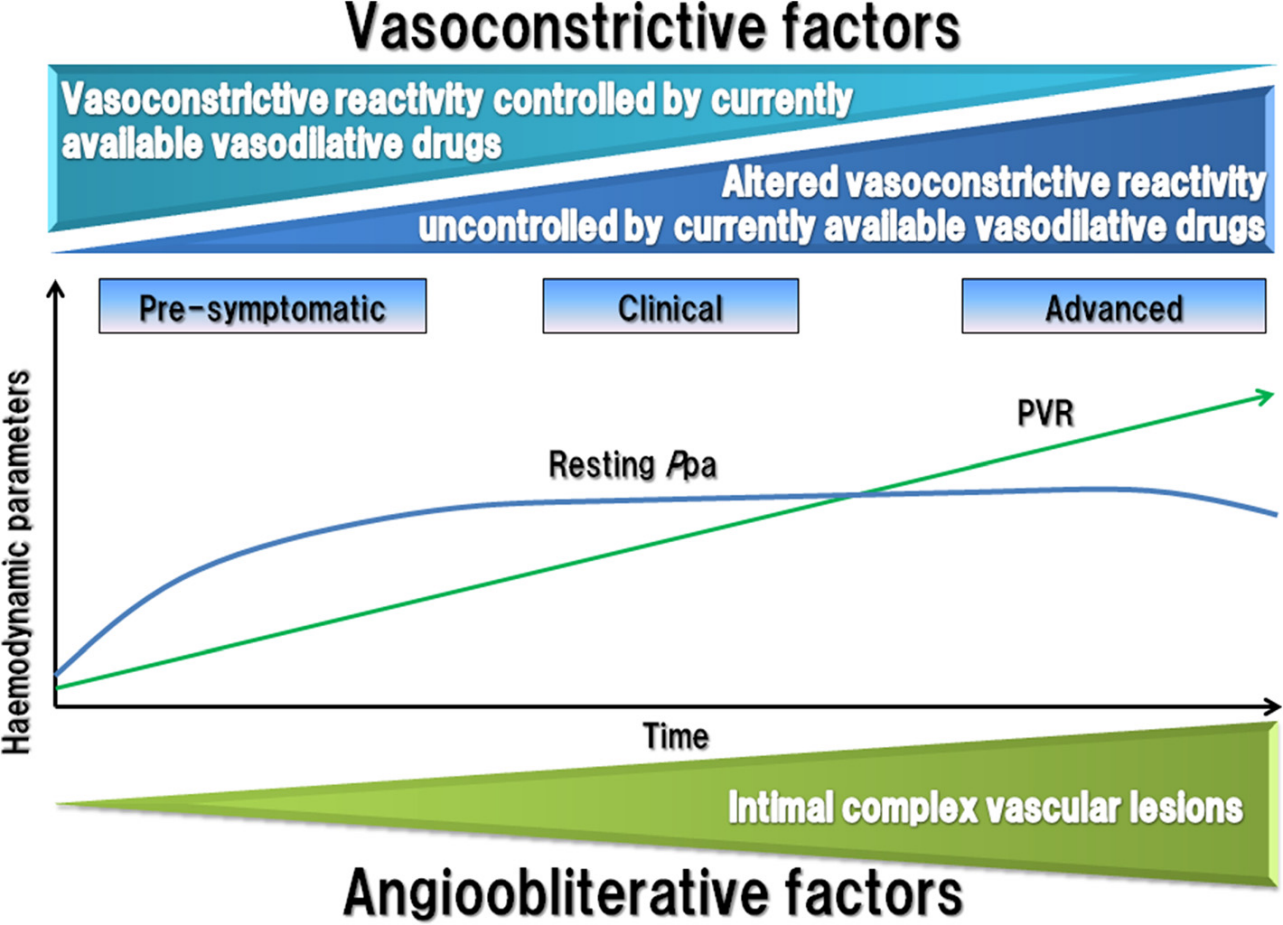
The determinants of severe pulmonary hypertension. The determinants of the elevation of the pulmonary arterial pressure in principle are vasoconstrictive reactivity which may be treatable with currently available vasodilator drugs. Fully developed and endstage disease may be characterized by complex vascular lesions and vasoconstriction that is refractory to treatment with presently available drugs. *P*pa: pulmonary arterial perssure, PVR: pulmonary vascular resistance

Almost five decades ago, the high pulmonary blood pressure associated with Heath-Edwards classification grades 1-3 was shown to be reversible following surgery that primarily eliminated the left to right shunt in PAH patients with CHD. However, PH associated with Heath-Edwards classification grade 4 was not reversible, at least acutely, after surgery [31]. These differences between grade 3 and 4 are due to the presence of complex intimal lesions that are composed of phenotypically altered mesenchymal-like cells [18, 22, 28, 29], indicating that the determinants of a fixed high pulmonary arterial pressure include complex vascular lesions, but not necessarily a high pulmonary blood flow. Oka et al. investigated the Sugen/Hypoxia rat model and found that pulmonary vascular tone remained to be an important contributing factor to the high *P*_pa_ even in the late stages of the disease. The role of increased vascular tone, in particular during exercise and perhaps also controlled by diurnal rhythms, may be underestimated in patients with fully developed angioobliterative disease [14] (Fig. 1). As the disease further progresses, a decrease in the cardiac output due to right ventricle dysfunction and failure results in a drop in *P*_pa_ while the pulmonary vascular disease is ongoing.

In the Sugen/Hypoxia model, the extent of medial thickness decreases gradually as the disease progresses, while the high pulmonary arterial pressure is maintained [15]. Thus, this finding in the animal model resembles the data reported by Stacher et al. These authors showed in their study that approximately 75 % of the lungs in patients with advanced PAH exhibited a medial fractional thickness which was similar to that in the controls, while 6/28 control lungs had an apparent medial fractional thickening not associated with plexiform lesions or PH [1]. As a reminder, in rodent models of PH induced by chronic hypoxia or monocrotaline injection, the medial remodeling and elevated pulmonary arterial pressure are potentially reversible [13].

Thus, we wonder whether medial remodeling is indeed the critical factor responsible for the increase in the pulmonary arterial pressure, or whether the medial wall thickening is to some degree an adaptation of the vasculature to intermittently higher shear stress. It has been suggested that PH alters the smooth muscle cells’ (SMCs) fiber length in order to maintain the transmural pulmonary arterial pressure as part of the pressure-adapting action of the pulmonary vasculature [32]. However, in human idiopathic PAH, the pulmonary arterial SMCs isolated from these patients showed dysfunctional voltage-sensitive potassium channels, which participate in pulmonary vascular tone control [33, 34]. It is therefore likely that these patients’ thickened pulmonary media layers, which are composed of functionally altered SMCs, have a direct role in increasing the pulmonary arterial pressure and that the degree of medial wall thickening is closely related to the vasoconstrictive potential [30].

Thus, it is likely that both medial and intimal wall remodeling contribute to the increase in pulmonary arterial pressure, as does adventitial remodeling due to proliferating fibroblasts [35] and deposition of extracellular matrix (ECM) in the pulmonary arteries [36]. Yet the degree of vasoconstriction may be limited in vessels altered by adventitial remodeling and in luminal stenosis (Fig. 1).

## Are there therapeutic strategies that are effective during different disease stages?

The elevated pulmonary arterial pressure appears to be driven not only by the vascular tone, but also by the phenotypically altered proliferating cells [24]. The currently available PH-targeted drugs including prostacyclin and its analogs, phosphodiesterase type 5 inhibitors and endothelin receptor blockers play a role both in opposing any abnormal vasoconstriction and in inhibiting the growth of SMC [37]. Despite therapy targeting PH, more than 30 % of PAH patients die within five years of receiving the diagnosis of severe PAH [38, 39]. The complex pulmonary vascular lesions remaining in the explanted lung tissue samples obtained from drug- treated PAH patients [1, 40] illustrate that the presently available drugs are not impacting the root causes of pulmonary vascular remodeling. Very differently, these drugs are effective in treating the PH in the monocrotaline rat model [41–44], which is characterized by medial thickening of the pulmonary arteries and muscularization of the pulmonary arterioles and the lack of intimal complex vascular lesions, including plexiform lesions [13]. This is likely attributable to the prominent role of pulmonary vasoconstriction in this monocrotaline model.

Early diagnosis is conceivably possible in certain forms of associated PAH (for example in patients with scleroderma, HIV/AIDS and sickle cell disease), however, it is unknown whether early disease stages in these patients cohorts are dominated by vasoconstriction. Indeed in patients with systemic sclerosis-associated PAH, the vascular stiffness caused by luminal stenosis with fibrous alterations and medial thickening of arterioles are prominent histopathological findings throughout almost all scleroderma disease stages [7]. This implies that early therapeutic interventions with vasodilator drugs may not impact the vascular lesions in the PAH patients with connective tissue disease.

Drugs which can de-remodel the severely altered and often occluded vessels and novel vasodilators which can cause vasodilation when the current vasodilator drugs are not effective are needed for the treatment of patients with severe PAH.

## Conclusion

Longitudinal studies of the Sugen/Hypoxia rat model of severe PAH have identified early and late disease stages [8, 9, 14, 15] and recent histopathological studies of lung explant tissues from patients treated with PH-targeting drugs have found only a tendency towards a correlation between the hemodynamic values and the variably represented pulmonary vascular lesions [1]. Figure 1 presents the hypothesis that in many, or in some, patients there may be a presymptomatic, initial disease stage where pulmonary vasoconstriction may be treatable with currently available drugs. However, it is not a foregone conclusion that early vasodilation treatment would prevent the progression to vasoobliterative disease. The mechanistic details of the interplay between hemodynamic pulmonary vascular stress, initial vascular cell apoptosis, subsequent exuberant cell growth and pulmonary arteriolar lumen obliteration remain elusive [24, 45].

## Abbreviations

PAH: Pulmonary arterial hypertension
IPAH: Idiopathic pulmonary arterial hypertension
Ppa: The mean pulmonary arterial pressure
CHD: Congenital heart disease
SUGEN5416: A vascular endothelial growth factor receptor blocker
RVSP: The right ventricular systolic pressure
PVR: Pulmonary arterial resistance
HPV: Hypoxic pulmonary vasoconstriction
COPD: Chronic obstructive pulmonary disease
ECs: Endothelial cells
PH: Pulmonary hypertension
SMCs: Smooth muscle cells
ECM: Extracellular matrix
HIV/AIDS: Human immunodeficiency virus/ acquired immunodeficiency syndrome
